# High-Resolution Structure and Internal Mobility of a Plant 40S Ribosomal Subunit

**DOI:** 10.3390/ijms242417453

**Published:** 2023-12-14

**Authors:** Olesya V. Kravchenko, Timur N. Baymukhametov, Zhanna A. Afonina, Konstantin S. Vassilenko

**Affiliations:** 1 Institute of Protein Research, Russian Academy of Sciences, 142290 Pushchino, Russia; olesyak@vega.protres.ru (O.V.K.);; 2 National Research Center, “Kurchatov Institute”, Akademika Kurchatova pl. 1, 123182 Moscow, Russia; baymukhametov.timur@gmail.com

**Keywords:** cryo-EM, plant ribosome, 40S ribosomal subunit, *Triticum aestivum*

## Abstract

Ribosome is a major part of the protein synthesis machinery, and analysis of its structure is of paramount importance. However, the structure of ribosomes from only a limited number of organisms has been resolved to date; it especially concerns plant ribosomes and ribosomal subunits. Here, we report a high-resolution cryo-electron microscopy reconstruction of the small subunit of the *Triticum aestivum* (common wheat) cytoplasmic ribosome. A detailed atomic model was built that includes the majority of the rRNA and some of the protein modifications. The analysis of the obtained data revealed structural peculiarities of the 40S subunit in the monocot plant ribosome. We applied the 3D Flexible Refinement approach to analyze the internal mobility of the 40S subunit and succeeded in decomposing it into four major motions, describing rotations of the head domain and a shift in the massive rRNA expansion segment. It was shown that these motions are almost uncorrelated and that the 40S subunit is flexible enough to spontaneously adopt any conformation it takes as a part of a translating ribosome or ribosomal complex. Here, we introduce the first high-resolution structure of an isolated plant 40S subunit and the first quantitative analysis of the flexibility of small ribosomal subunits, hoping that it will help in studying various aspects of ribosome functioning.

## 1. Introduction

Ribosome is a molecular machine that translates genomic information into the amino acid sequence. This huge nucleoprotein complex with a molecular weight of 2.5–4 MDa consists of two unequal subunits: “small”, referred to as 40S in eukaryotes and 30S in prokaryotes and organelles, and “large”, named 60S or 50S, respectively, where “S” stands for Svedberg sedimentation coefficient. While the large ribosomal subunit functions only as a part of a whole ribosome, participating in the elongation of a protein chain, the small subunit has extra functions, being responsible for the initiation of translation and for the recycling of ribosomes after termination. Such versatility makes the small ribosomal subunit a tempting object to study.

The detailed structure of the bacterial ribosome was first resolved more than 20 years ago using X-ray analysis [1], and since then, a plethora of structural data have been obtained for both prokaryotic and eukaryotic ribosomes from different sources (see [2] for the complete list of structural studies). In parallel, the cryo-electron microscopy (cryo-EM) approach for structure reconstruction has evolved, where freezing the object in amorphous ice avoids the need for the extremely complex and labor-consuming crystallization stage. The last decade has been a breakthrough for cryo-EM analysis, especially in the study of large molecular complexes. The drastic enhancement in the resolution has made cryo-EM a preferred method in many research areas [3].

Ribosomes and ribosomal subunits are considered ideal objects for single particle cryo-EM analysis [4], as they possess homogeneity, internal stability, pronounced characteristic shape and convenient size, which is sufficient for high-resolution reconstruction without requiring excessive computing. All this made ribosomes the most popular target for cryo-EM structural analysis.

However, a ribosome as an object for cryo-EM study has certain drawbacks. First, it is a huge nucleoprotein complex consisting of 3–4 long RNA chains and 70–90 proteins. Despite the rather conservative structure of their internal parts, ribosomes reveal a lot of variations in both sequence and structure of their peripheral regions, especially in rRNA expansion segments and the N- and C-termini of eukaryotic ribosomal proteins. Because of significant heterogeneity and lack of symmetry, building a ribosome atomic model from scratch takes a real investment of time and effort. This is why researchers today tend to study different states and complexes of ribosomes from only a few species, for which basic models have already been constructed. For example, the structures of ribosomes from only four plant species were solved at a high resolution, including two cytoplasmic [5,6], one chloroplastic [7], and one mitochondrial [8], and among 334 atomic models of isolated small ribosomal subunits (SSUs), there is not one of plant origin [2].

Another problem is that some peripheral regions of ribosomal subunits are quite flexible, although the core parts are relatively stable, allowing one to achieve an amazing 1.5 Å local resolution. The most prominent mode of internal mobility of the small ribosomal subunit is the relative movement of its “head” and “body” domains, usually referred to as “swiveling” [9] and “tilting” [10] of the head (see [2] for a detailed definition). The processing of the subunit as a solid body results in poor resolution of the head domain. This problem of large-block internal movement has been successfully solved through use of the multibody, or local, refinement technique [11,12]. This method involves the application of isolating masks to parts of the electron density belonging to more or less separated domains, which are considered independent particles in further processing. This approach allows to obtain uniformly high resolution throughout the inner part of the 40S ribosomal subunit map. Lately, this method has become a standard for cryo-EM analysis of small ribosomal subunits.

Unfortunately, the resolution of smaller flexible peripheral parts of the ribosome structure, such as rRNA expansion segments and surface-exposed domains of ribosomal proteins (RPs), cannot be substantially improved by using a masking technique. In this case, the size-to-resolution ratio for isolated ribosomal parts is too small for effective processing by particle search and alignment algorithms.

Recently, an alternative approach has been introduced, namely 3D Flexible Refinement (3DFlex) [13], which enhances cryo-EM analysis by using a deep learning algorithm that captures the conformational variability in an object. The method is based on a deep neural network model that describes conformational variability in terms of a parameterized latent space of deformation fields that encode flexible motion of a high-resolution 3D density map. The obtained set of parameters and latent coordinates allows one to study a flexible object’s motion over its conformational landscape and enables the building of a high-resolution electron density map.

Here, we focused on a cryo-EM analysis of the cytoplasmic small ribosomal subunit obtained from wheat germ extract, the most popular system for in vitro study of translation in plants. We report a high-resolution (2.3–2.5 Å) cryo-EM reconstruction of the *Triticum aestivum* (common wheat) 40S ribosomal subunit. A detailed atomic model was built, including the majority of the rRNA and some of the protein modifications. We used the cryo-EM dataset to train the 3DFlex algorithm and identify the modes of 40S subunit flexibility. The population of all the conformational states of the free 40S subunit was determined. The analysis revealed three weakly coordinated major motions of the head domain and the prominent shift of an expansion segment 6d of the rRNA helix h21. It turned out that the relative movement of 40S domains is more complex than previously thought. In addition, taking into account the subunit flexibility helped to recover high-resolution details in regions blurred in the conventional cryo-EM map. Our study provides the first quantitative analysis of the internal mobility of a small ribosomal subunit and the first high-resolution structure of a monocot plant 40S subunit.

## 2. Results

### 2.1. Structure of the 18S rRNA of a Monocot Plant

**Figure 1:**
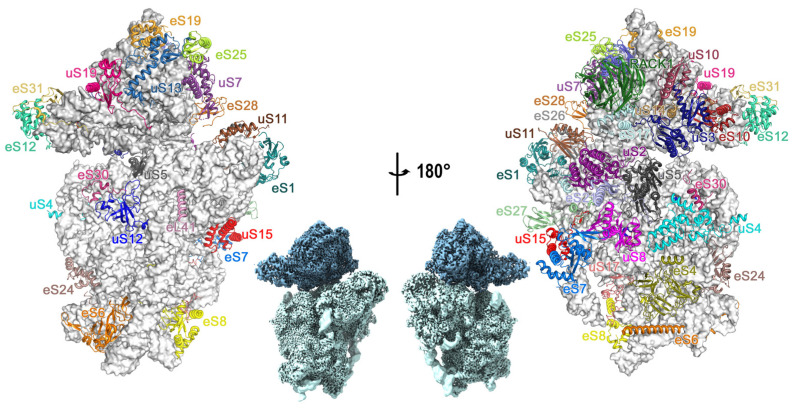
The newly constructed atomic model for the *Triticum aestivum* ribosomal 40S subunit. The rRNA is represented as grey atom spheres and individually colored ribosomal proteins are shown as ribbons. The insets depict the corresponding projections of the stacked head and body density maps.

By implementing the multibody refinement algorithm (Supplementary Figure S1), we obtained cryo-EM reconstructions of both the head and body domains with nominal resolutions of 2.55 Å and 2.34 Å, respectively, extended to 2.0–2.2 Å in the most stable internal regions (Supplementary Figure S2). This allowed us to build a precise atomic model with a MolProbity score of 1.72 (Figure 1).

**Figure 2:**
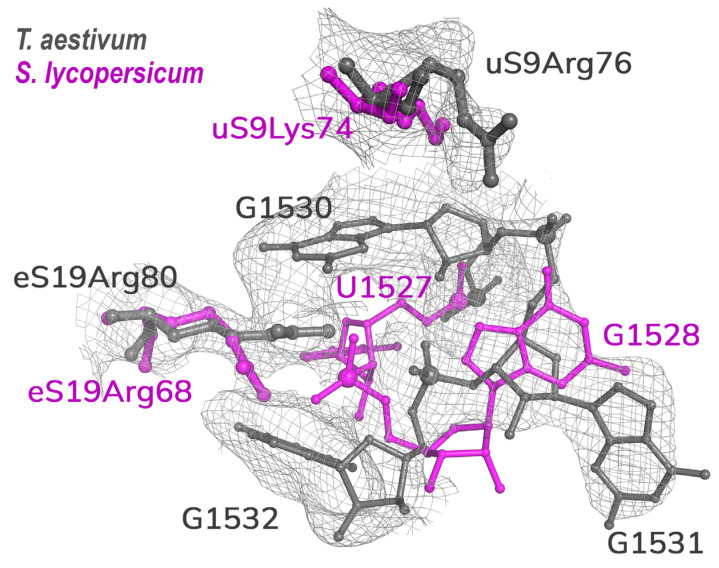
An example of differences in the structure of 18S RNA from monocot and dicot plants. An additional G nucleotide in the head domain of wheat rRNA alters its interaction with the ribosomal proteins.

An 18S rRNA sequence with GenBank accession code XR_006452643.1 was selected as the best conforming to the electron density map (Supplementary Figure S3). It is the only *Triticum aestivum* rRNA sequence that was checked through direct rRNA sequencing. Interestingly, a nucleotide corresponding to G1531 is absent in the 18S rRNAs of tomato and tobacco ribosomes, and the relevant part of rRNA models reveals significant rearrangements (Figure 2). This deletion might be compelled by the need for tight interaction with N-terminal parts of eS19 and uS9 ribosomal proteins, which exhibit divergent species-dependent amino acid sequences.

With the data collected, we were able to construct an enhanced model of plant 18S rRNA secondary structure (Supplementary Figure S4). The inspection of the precise 3D model allowed us to identify a lot of non-canonical base pairs, clarifying the endpoints of helical regions.

### 2.2. Identifying the Correct Sequences of Paralogous Ribosomal Proteins

The first cryo-EM analysis of wheat ribosome was performed more than 10 years ago [14]. At the time, the majority of *Triticum aestivum* genes had not yet been sequenced, and protein sequences from *Oriza sativa* and even yeast were used to build the models of certain wheat ribosomal proteins. The relatively low resolution of the density map obtained at the time (5.5 Å) did not allow for validating such substitutions reliably.

Since then, plenty of additional genomic information has emerged, and additional sequences of *Triticum aestivum* RP genes have become available. Plant ribosomal proteins are usually encoded by 2–6 paralogous genes [15]. The high resolution of the density maps allowed us to perform “back sequencing”—the evaluation of nucleotide and amino acid sequences based on their conformity with the corresponding electron density. In most cases, we were able to unambiguously choose one of the paralogs that most adequately fitted in the map (Supplementary Table S2). This indicates that the distribution of proteins of varying sequences among ribosomes can be uneven and that one of the paralogs is preferable, at least at given growth conditions.

Interestingly, the most protruding “beak” part of the head domain is much better resolved in the map of the isolated plant 40S subunit than in a complete 80S ribosome, free [5] or translating [6]. This allowed us to significantly enhance the structure of ribosomal proteins eS12 and eS31 and to clarify their interactions with 18S rRNA and each other.

**Figure 3:**
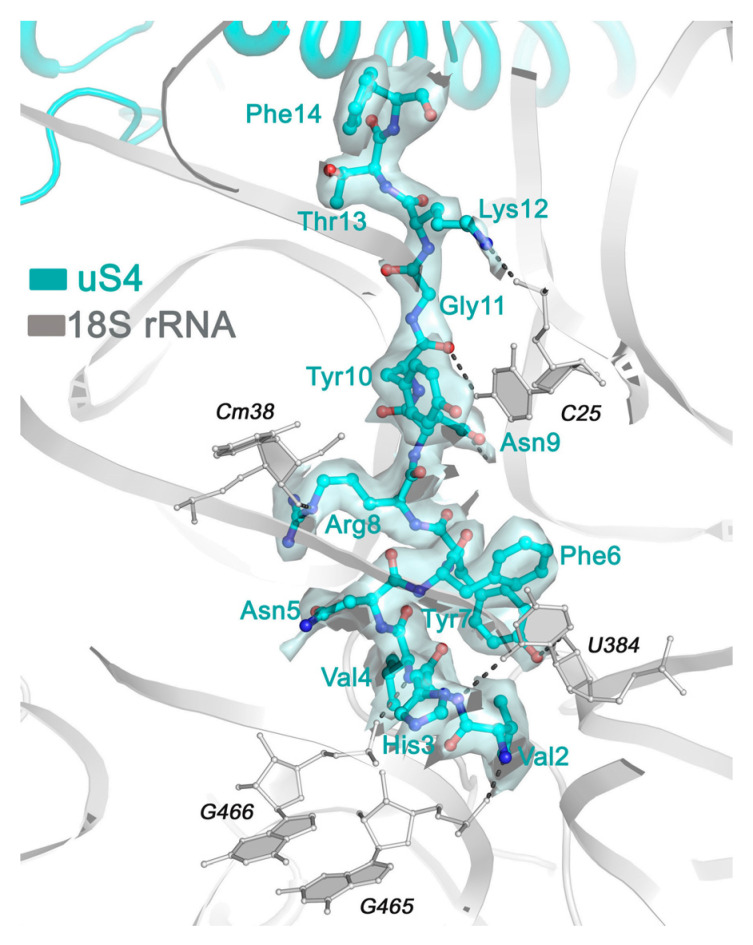
An example of a ribosomal protein sequence selection based on its fit to the density map. A specific N-terminal segment allowed the unambiguous selection of a uS4 sequence from a set of homologs.

Despite the presence in the database of several *Triticum aestivum* paralogous genes of highly conservative ribosomal protein uS4, the only sequence with an N-terminus fitted decently into the relevant part of the density map is the UniProt entry W5FPA7, attributed to a chloroplast S4 ribosomal protein (Figure 3). The corresponding gene is located on nuclear chromosome 3 and can code for cytoplasmic ribosomal protein. Most likely, this is the case of the wrong gene characterization.

The more ambiguous situation arose in the case of very short protein eL41, virtually a peptide, which is situated at the interface side, binding both the large and small ribosomal subunits. We were unable to find even a roughly adequate sequence within the *Triticum* genomes, neither by description nor by homology search. Such a lacuna in the data forced us to use a rice sequence as a substitute that fitted perfectly in the map and should not be much different in the closely related plant.

**Figure 4:**
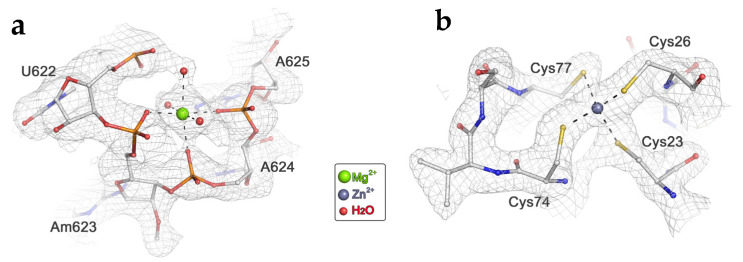
High-resolution features resolved in the 40S density map. (**a**) Fragment of the wheat 18S rRNA model, including a Mg^2+^ ion with octahedral coordination. (**b**) Zinc finger structure in the protein eS26 atomic model. The grey mesh depicts the electron density of the wheat 40S cryo-EM map.

We also modeled the solvation of the wheat 40S ribosomal subunit by locating water molecules, magnesium (Mg^2+^), zinc (Zn^2+^) and monovalent ions (Figure 4).

### 2.3. Visualization of Post-Transcriptional Modifications of the 18S rRNA

The high resolution of our reconstruction of the wheat 40S subunit enabled us to analyze post-transcriptional and post-translational modifications of 18S rRNA and RPs and elucidate their role in ribosome structure formation.

Methylation of 2′-OH ribose (2′-O-Me, Nm) and pseudouridylation (Ψ) are the most abundant post-transcriptional modifications (PTM) of rRNA in eukaryotes [16,17].

Unfortunately, in a cryo-EM map with resolution of worse than 2 Å, it is impossible to detect ribose methylation confidently without confirmation by independent biochemical data. Systematic studies of rRNA methylation profiles of *Arabidopsis thaliana* [18,19,20] and *Solanum lycopersicum* [2], both dicots, published previously, provide a solid base for the structural data analysis.

If the methyl group is not fixed by specific interactions, its rotation around the C^2′^–^2′^O axis can blur its density in the analyzed map. Another difficulty might be the low modification level of some PTMs. In this case, the methyl group electron density of the averaged map can be too weak to make a confident conclusion about the existence of modification. However, according to [5], only about 4% of plant 18S rRNA modifications reveal stoichiometry less than 40% and only 9%—less than 70%. Therefore, the low abundance of certain modifications is not a very important issue in structural PTM analysis.

An even more uncertain situation is the case of pseudouridinilation as far as the electron density of Ψ base is indistinguishable from those of uridine at any accessible resolution. It is, however, helpful that pseudouridine has an additional hydrogen bond donor N^1^ (C^5^ in uridine) that can bond to a suitable proton acceptor. In view of this, the presence of a water molecule or an acceptor group in the plane of suspected Ψ base at a distance of less than 3 Å from imino nitrogen N^1^ [21] is considered to be strong evidence in favor of the modification.

**Figure 5:**
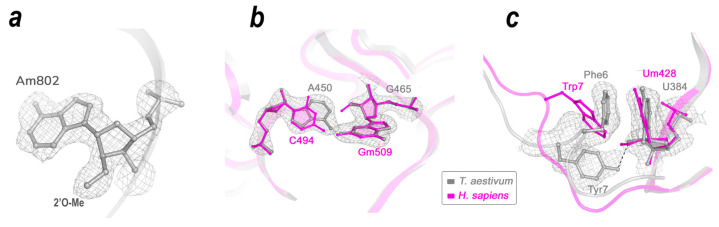
Effect of 2′-O-methylations on the structure of an rRNA. (**a**) An example of a methylated adenosine fitted into the experimental cryo-EM map of *Triticum aestivum* SSU. (**b**) Methylation of ribose supports the local structure of the rRNA chain by filling the empty space. (**c**) Modification of the rRNA tunes its interaction with a species-specific chain of uS4 ribosomal protein. Comparison of the corresponding parts of human (magenta) [22] and wheat (grey) 18S SSU atomic models. The grey mesh depicts the electron density of the wheat 40S cryo-EM map.

Visual analysis of the 18S rRNA-related part of the cryo-EM density maps with due regard for local resolution and taking into account published biochemical data allowed us to confirm 30 2′-O-Me and 32 Ψ modification sites out of 44 and 42 reported in biochemical studies, respectively (Figure 5a, Supplementary Tables S3 and S4). In addition, we found seven characteristically shaped regions in the electron density map that could be attributed to 2′-O-Methyl groups not previously reported for plant ribosomes. For two 2′-O modifications clearly visible in tomato ribosome map [5], corresponding methylation densities were not detected in the wheat 40S map, despite a high local resolution. These modifications can be provisionally considered absent in the ribosome of monocot plants. The presence of electron densities of acceptor groups located at proper positions relative to presumed N^1^ H-bond donor allows one to consider 10 uridines as putative pseudouridines that were not described previously.

Comparison of various atomic models of ribosomal subunits in the context of rRNA modification profiles could clarify the role of the modifications in the formation of ribosome structure. As an example, in human 18S rRNA, the base of C492 interacts with the modified ribose of Gm509 [22], while in wheat, it is A450 at the same spatial position that rests against the corresponding G465 ribose (Figure 5b). Here, the substitution of C for a larger A base has a very minor structural effect since the methyl group at 2′-O-Me of Gm509 fills the empty space and the structure of the sugar-phosphate backbones remains fairly similar.

Also, variability in rRNA modification profiles can be important to secure effective binding to certain parts of ribosomal proteins, especially highly variable eukaryote-specific N- and C-terminal extensions [23] that serve as anchors attaching proteins to ribosomal RNAs. For instance, 2′-O of Um428 in human 18S rRNA is methylated, while corresponding ribose in wheat (U384) remains unmodified, therefore being able to form an H-bond with the OH-group of TYR7 in plant-specific 5′-end of uS4 protein. This binding provides a sharp kink in the protein chain that ensures the perfect stacking interaction between PHE6 and the U384 base (Figure 5c).

Seven of the detected pseudouridines are a part of Ψ–U and Ψ–Ψ base pairs, and three are adjacent to U-U pairs (see Supplementary Figure S4). It was shown that replacement of U with Ψ stabilizes RNA duplexes, especially in the case of non-canonical base pairing [24]. In addition, 20 stabilized Ψ–A and Ψ–G base-pairs found near the ends of rRNA helices can increase their stability.

We detected modifications that are highly conserved in eukaryotes and were recently finally proved to be present in plants [5]: two N^6^,N^6^-dimethyl-adenines (m^6^_2_A1792, m^6^_2_A1793), two N^4^-acetyl-cytidines (ac^4^C1285, ac^4^C1784), N^7^-methyl-guanine (m^7^G1584) and N^6^-methyl-adenosine (m^6^A1774). We also found that the density of hypermodified N^1^-methyl-N^3^-(α-amino-α-carboxypropyl)-pseudouridine (m^1^acp^3^Ψ1196) is present in the wheat germ 18S rRNA map, while previously, it was reported that it was not detected in etiolated germinating wheat seeds [25]. The authors supposed that the absence of this modification is due to the development of seedlings in the dark and the modification reaction is to be light-induced. The fact that we detected this modification in dry seeds formed under normal lighting conditions does not negate this assumption.

### 2.4. Post-Translational Modifications of Ribosomal Proteins

**Figure 6:**
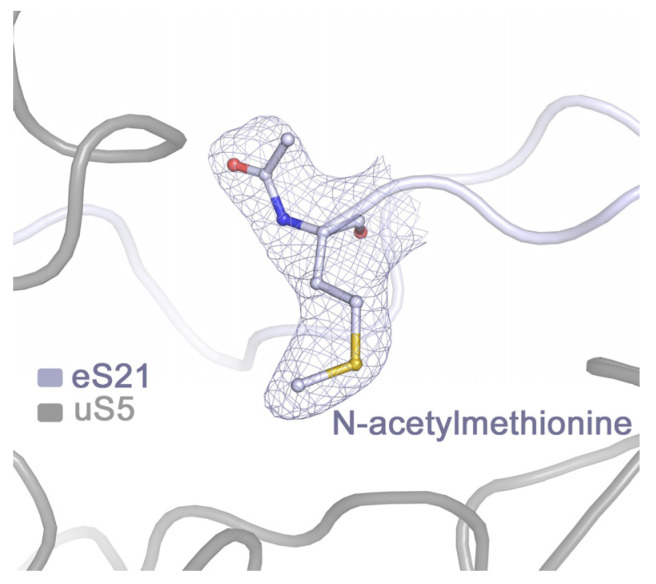
N-terminal acetylation of the eukaryote-specific ribosomal protein eS21.

Modifications of ribosomal proteins in plants are mostly limited to initiator methionine removal, N-terminal acetylation, and phosphorylation [26,27]. Unfortunately, most N-termini of eukaryotic RPs are very flexible, and their electron density is absent or blurred in the cryo-EM map. We succeeded in proving the acetylation of only one N-methionine in the protein eS21 (Figure 6). Resolved electron density of 12 proteins started from the second amino acid, and for 9 of them, a tightly packed N-terminus allowed us to draw a valid conclusion on the post-translational methionine removal. As for phosphorylated serines, they are all located in C-terminal parts of RPs not resolved in the cryo-EM map.

In addition, as in the case of tomato ribosome structure [5], an extra density in the backbone of Asp137 in ribosomal protein uS11 indicates its conversion to isoaspartate (isoAsp) via dehydration followed by hydrolysis.

### 2.5. The 40S Subunit Reveals Various Modes of Internal Mobility

**Figure 7:**
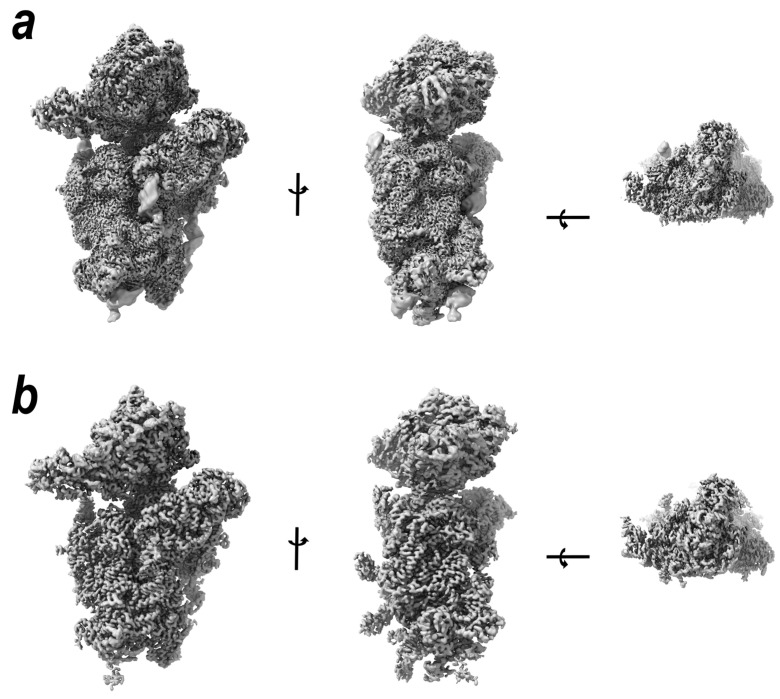
3D Flexible Refinement reveals more details in unstable regions. (**a**) Projections of the combined head and body cryo-EM reconstructions received by means of local refinement. (**b**) Projections of the wheat full-size 40S density map obtained using 3DFlex refinement algorithm.

By implementing the novel 3D flexible refinement algorithm [13], which takes into account the deformation of the structure across the conformational landscape, we obtained a reconstruction of the full-size 40S subunit at nominal resolutions of about 2.68 Å (Figure 7). The most noticeable effect of the 3DFlex processing is the more even electron density values for various structural elements. This is particularly noticeable in the case of rRNA, where the sugar-phosphate backbone generally reveals higher mobility compared to the bases. The obtained density map was used to improve the model in some intrinsically flexible regions. We succeeded in filling several gaps in the models of both rRNA and protein chains.

Interestingly, the local resolution of the stable regions in the internal body parts of the 3DFlex map appeared to be close to that the local refinement map (Supplementary Figure S2). However, even visual inspection of the maps revealed that the latter better represents fine details, like modifications and heteroatoms. The causes of this discrepancy lie in the peculiarities of the Fourier shell correlation algorithm that is, basically, a comparison of two independently built half-maps and the estimation of the local deviations. Since the 3DFlex algorithm is designed to diminish the differences between similar projections, it, thereby, diminishes the differences between two averaged half-maps. But this works only for the details with the size that exceeds physical resolution. The details of the smaller size are ignored by the algorithm and, therefore, can be lost in the transformation procedure, which, however, gives a map with formally similar local resolution values.

3DFlex describes conformational variability as a movement in a parameterized latent space of deformation fields encoding flexible motion [13]. Latent coordinate vectors specify positions of each conformation over the conformational landscape. The more complicated the deformation motions inside the object are, the more latent coordinates are needed to describe them reliably. Simulation of a density map movement along each latent component allows us to visualize the major motions inside the flexible structure of the object of interest, the plant 40S ribosomal subunit in our case.

**Figure 8:**
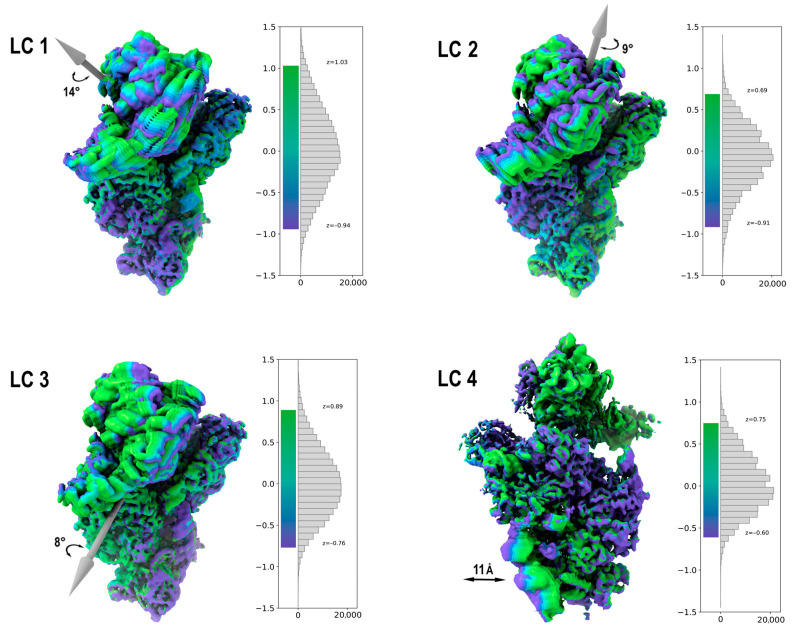
Internal mobility of the plant 40S ribosomal subunit. The stroboscopic representation of the intermediate maps depicting a continuous motion along a given latent coordinate (LC). Grey arrows represent the rotation axes. Black arrows define the range of the motion. Color bars indicate the population of the intermediate conformations.

We used up to five latent dimensions for the 3DFlex algorithm training and built 41 intermediate frames (maps) for each coordinate to visualize the continuous structural heterogeneity (Figure 8). It was found that the motion along the fifth coordinate is insignificant comparing to the first four, reflecting just the minor changes in the 40S structure. This means that four latent coordinates are enough for the reliable description of the 40S internal mobility.

The first three latent coordinates correspond to the mobility of the head domain. Coordinates 1 and 2 match swivel motion, while coordinate 3 defines as tilting. The bimodality of the swivel motion makes it clear that in the case of an isolated 40S subunit, rotation of the head domain is a complex two-dimensional process. Correlations between these motions reflect their interrelation, e.g., a mutual influence level (Supplemental Figure S5). A recent statistical study [2] revealed that the head domain of SSU as a part of a complete ribosome, free or translating, exhibits a complex range of orientations involving rotation and tilting. It was shown that the head rotation angles are strongly correlated with the tilt angles. Here, we demonstrated that in the case of the isolated 40S subunit, the various modes of head motion are weakly correlated (Supplementary Figure S5). This means that it is not internal restraints that provide coordination of relative positions of SSU domains; rather, it is a result of the specific interactions with 60S subunit, translation factors, tRNAs and mRNA. The 40S subunit itself is flexible enough to adopt any conformation required at a given state of the translation epicycle. At the same time, the structures of isolated late intermediates of bacterial SSUs reveal a range of characteristic head positions [2] that can be a reflection of the fact that not completely formed rRNA structure and variations in protein composition can fix a specific head-to-body conformation and so impede the interaction of SSU with a large ribosomal subunit.

Interestingly, the fourth latent coordinate is not related to the mobility of the head domain but describes the motion of the ES6d 18S rRNA expansion segment, sometimes referred as a “right foot” (Figure 8). Unlike the first three coordinates describing mobility of the head domain, the major part of this motion is not a rotation around a certain vector but basically a rigid-body 11 Å shift of a significant part of the rRNA chain, coupled with only 1.5° turn. To allow for such motion, the structure of helix 21 must undergo notable rearrangements. Analysis of the intermediate density maps describing the transition along the fourth coordinate revealed that the C-terminus of ribosomal protein eS6 remains bound to the ES6d segment, and its very long α-helix can act like a spring to provide this unusual motion.

## 3. Discussion

Over the past few years, cryo-electron microscopy, especially microscopy of ribosomes, has made impressive progress. Five years ago, 5 Å resolution was considered quite decent in the structural analysis of ribosome and ribosomal subunits, whereas, today, 2.0–2.5 Å is a standard, in some cases reaching even 1.5 Å resolution, previously accessible only for small molecules. Cryo-EM has grown to become a real alternative to X-ray analysis. This breakthrough is attributed to an increase in computing power and the development of new data processing algorithms, although EM-hardware improvements, such as new fast data collection systems, have also played a role. Comprehensive classification, local refinement, and DeepEMhancer post-processing have greatly enhanced and simplified the process of obtaining high-resolution data. What used to take weeks can now be accomplished in just a few hours. But the avalanche of new cryo-EM data is held back by the fact that the building of atomic models is still a time-consuming semi-manual process. Therefore, every new high-resolution structure of ribosomes or subunits from different orders, families, genera, or even species is important as it broadens the experimental base for further structural and functional studies.

Here, we provide our contribution by presenting the first high-resolution structure of the isolated 40S subunit of a plant ribosome. This input is significant as only two high-resolution (~2.5 Å) [5,6] and one medium-resolution (5.5 Å) [14] structures of the cytoplasmic plant ribosomes are currently available. We conducted a comprehensive structural study of the *Triticum aestivum* (common wheat) SSU, identified the best-suited paralogs of the 18S rRNA and ribosomal protein genes, and described notable deviations from the structure of a dicot plant ribosome.

The rRNAs and ribosomal proteins are decorated with various post-transcriptional and post-translational modifications. Direct visualization of such modifications can provide a mechanistic understanding of their role in the formation of ribosome structure. The analysis of PTMs in the wheat 40S subunit allowed us to make plausible assumptions regarding the distinctiveness of the 18S rRNA modification profile in monocot plants. The comparison with the atomic model of the markedly dissimilar human 40S subunit yielded data indicating an important structural role of rRNA modifications, which still remains a matter of debate.

The 40S subunit is involved in all stages of protein biosynthesis, spanning from initiation to recycling. The flexibility and mobility of its specific parts, particularly the head domain and rRNA expansion segments, were shown to be functionally significant. It was demonstrated that the position of the head relative to the body varies in different translation complexes and ribosome intermediate states [2]. Some other mobile elements can also be fixed in certain conformations, such as the rRNA helix 44 when being engaged in the binding of the 60S subunit, or ES6a expansion segment that was shown to interact with the b subunit of the eIF3 initiation factor upon formation of the preinitiation complex [28]. All of these make the study of the flexibility of a small ribosomal subunit an important fundamental scientific task.

Here, we performed a quantitative analysis of the internal mobility of the plant 40S ribosomal subunit. We defined four weakly dependent motions describing its internal mobility and determined their boundaries. It was shown that the rotation of the SSU head is not bimodal, as was thought before [2], but rather trimodal, and the overall movement of the head domain can be rather complex. Furthermore, we described a significant translational motion of a eukaryote-specific 18S rRNA expansion segment that may also be functionally important.

The obvious limitation of a free small ribosomal subunit as a research subject is that in the cytoplasm, it is always bound to a large subunit or recruits specific translation factors. But it is these immutable homogenous “classical” subunits that serve as scaffolds for all the translational machinery, and we expect the data we presented here to be helpful in studying various aspects of small ribosomal subunits functioning and in building models of interactions with other components of the translational apparatus.

## 4. Materials and Methods

### 4.1. Cryo-EM Sample Preparation

In this study, 500 µL of wheat germ extract (WGE) 300 OD/mL prepared as described in [29] from Moskovskaya wheat variety was dialyzed overnight against 200 mL of 5/500 buffer (20 mM HEPES/KOH pH7.5, 500 mM KCl, 5 mM MgCl_2_, 2 mM DTT) at 4 °C. The sample was loaded at 28 mL 5–30% linear sucrose gradient (5/500 buffer) and centrifuged in SW28 bucket rotor, Optima centrifuge (Beckman Coulter, Brea, CA, USA), for 18 h at 4 °C. The gradient was fractionized into 200 µL aliquots, photometrized at 260 nm, and the fractions belonging to the top major peak were joined and dialyzed overnight against 200 mL of 5/100 buffer (20 mM HEPES/KOH pH7.5, 100 mM KOAc, 5 mM Mg(OAc)_2_, 2 mM DTT) at 4 °C. The sample was concentrated in Centricon YM (Merck Millipore, Burlington, MA, USA), adjusted with 5/100 buffer to the optical density of 36 OD_260_ (~2.5 µM 40S) optimal for storage, aliquoted, frozen in liquid nitrogen and stored at −80 °C. Purity of the preparation was checked by SDS-PAGE through the comparison with the standard 40S protein profiles.

Quantifoil R1.2/1.3 300 mesh grids, coated with 2 nm amorphous carbon film, were glow-discharged for 30 s at 15 mA using PELCO easiGlow system (Ted Pella, Redding, CA, USA) prior to sample application. Then, 3 µL of 40S subunit preparation adjusted to 0.6 µM concentration with 5/100 buffer was applied to the grids, blotted for 3 s at 10 °C and 100% humidity, and plunge-frozen in liquid ethane using Vitrobot Mark IV (Thermo Fisher, Waltham, MA, USA).

### 4.2. Cryo-EM Data Collection and Processing

Data were acquired using a Titan Krios transmission electron microscope (Thermo Fisher Scientific, Waltham, MA, USA) equipped with a field-emission electron gun (X-FEG, Thermo Fisher Scientific, Waltham, MA, USA), a spherical-aberration corrector (CEOS GmbH, Heidelberg, Germany) operated at 300 kV, and a Falcon II direct electron detector (Thermo Fisher Scientific, Waltham, MA, USA). A total of 6201 image stacks (movies) were acquired at a nominal magnification of 75,000× (calibrated pixel size of 0.86 Å) and in a nominal defocus range from −0.6 to −1.8 µm using the automated data acquisition software EPU ver. 1.9.1.16 (Thermo Fisher Scientific, Waltham, MA, USA). The detailed data acquisition and processing parameters are listed in the Supplementary Table S1.

The collected cryo-EM data were processed using the following sequence: pre-processing (Warp ver. 1.0.9 [30])—preliminary classification and refinement (cryoSPARC ver. 4.2.1 [31])—Bayesian polishing (RELION ver. 4.0.1 [32,33])—Local and 3D Flex refinement (CryoSPARC)—post-processing (DeepEMhancer [34]) (see Supplementary Figure S1 for the schematic representation).

The acquired images were semi-automatically inspected using assessment criteria—thresholds for defocus (<2 µm), estimated resolution (<3 Å) and drift (<2 Å). Images with an astigmatism of an estimation greater than 100 nm were discarded. Thus, 5521 images containing 982,063 extracted particles (box size 480 px) were selected for further data processing.

Multiple rounds of 2D classification were performed to maximize the number of true-positive particles and remove non-SSU objects. To minimize negative preferred orientations, hetero-refinement was employed. A resulting subset of 40S particles was used for ab initio reconstruction of the initial reference consensus map. Hetero-refinement [11] together with per-particle defocus adjustment and per-exposure-group adjustment of higher-order CTF terms gave a 2.7 Å resolution consensus map.

Therefore, 256,182 selected particles were subjected to Bayesian polishing, an approach that allows for per-particle, reference-based correction of beam-induced motion, performed directly without any training step using default parameters (𝜎vel = 0.2, 𝜎div = 5000, 𝜎acc = 2). The final reconstruction of the total 40S subunit yielded the reference map with an estimated resolution of 2.44 Å (sharpening B-factor: m77.4 Å^2^) based on the gold-standard Fourier Shell Correlation (FSC) 0.143 cut-off [35,36].

For the separate high-resolution reconstructions of the head and body domains, two soft masks outlining each domain were created in Chimera software ver. 1.17.3 (UCSF) on the basis of the consensus map and used as an input for the local refinement procedure. The resolution of the head domain reconstruction was estimated as 2.55 Å (B-factor: −86.6 Å^2^). Additional application of the Bayesian polishing increased the resolution of body domain from 2.44 A to 2.34 A (B-factor: −76.0 Å^2^).

Local filtering of all maps was performed using the estimation of local resolution (local windowed FSC method [37], cryoSPARC). Resulting density maps were post-processed by DeepEMhancer procedure using a pre-trained tightTarget model.

### 4.3. Model Building

PDB structures 7QIX and 7QIY (cryo-EM reconstruction of *Solanum lycopersicum* 40S ribosomal subunit body and head domains) were used as references for the modeling of *Triticim aestivum* 40S subunit body and head domains, respectively. Models of both domains were fitted into corresponding cryo-EM maps as a rigid body using Chimera software ver. 1.17.3 and ChimeraX ver. 1.5 (USCF). Protein and rRNA chains were visually inspected in WinCoot (ver. 0.9.8.93) and manually fitted into the density map. The resulting models were built by several rounds of real-space refinement in the Phenix program suite and manual adjustments in WinCoot. For model validation, we used MolProbity tool (Phenix ver. 1.20.1-4487, Lawrence Berkeley Laboratory, Berkeley, CA, USA). Model-building parameters and refinement statistics are summarized in Supplementary Table S1.

### 4.4. 3D Flexible Refinement

The CryoSPARC version of a motion-based neural network model approach, 3D Flexible Refinement (3DFlex) [13], was used. To reduce computational time, the final subset of particles was downsampled from 480 px to 440 px by Fourier cropping (Nyquest resolution ~1.9 Å) and a new consensus map was built. For the training of the 3DFlex model, the input particles were further downsampled to 200 px. A tetrahedral mesh was generated using default parameters. For the model training, 5 latent dimensions were used, 1 additional final training epoch was added, and the latent centering strength parameter was increased to 40. In the 3DFlex reconstruction, the default number of iterations was used, resulting in a map with 2.61Å resolution. Increasing the number of iterations led to a deterioration of the resolution calculated using the FSC = 0.143 threshold, while diminishing the number led to an insufficient convergence, as reflected in the distortion of the FSC curves at high frequencies. To visualize the continuous structural heterogeneity, five sets of 41 frames (maps) were built, reflecting the motion along each of 5 latent coordinates (zi) around the mean point of the corresponding conformational distribution in the ranges of z1: [−0.94; 1.03], z2: [−0.91; 0.69], z3: [−0.76; 0.89], z4: [−0.60; 0.75], z5: [−0.69; 0.61].

## Data Availability

The cryo-EM maps have been deposited in the EMDataBank (EMDB codes: 18951 for 40S body, local refinement; 18903 for 40S head, local refinement; and 18911 for total 40S subunit, 3DFlex refinement). The coordinates for the atomic models have been deposited in the Protein Data Bank (PDB codes ID 8R6F for 40S body; ID 8R57 for 40S head).

## Supplementary Materials

The following supporting information can be downloaded at: https://www.mdpi.com/article/10.3390/ijms242417453/s1. References [38,39] are cited in the supplementary materials.
