# Supplementary material for: High-Resolution Structure and Internal Mobility of a Plant 40S Ribosomal Subunit
Source: Int J Mol Sci. 2023 Dec 14;24(24):17453. doi: 10.3390/ijms242417453 (PMC10743738; doi:10.3390/ijms242417453)
Supplement: Supplementary file 1 [file ijms-24-17453-s001.zip › Kravchenko_et_al_SupplementaryMatherials.pdf]

Supplementary materials

High-resolution structure and internal mobility of a plant 40S ribosomal subunit

Olesya V. Kravchenko [1], Timur N. Baymukhametov [2], Zhanna A. Afonina [1] and Konstantin S. Vassilenko [1,*]

[1] Institute of Protein Research, Russian Academy of Sciences, 142290 Pushchino, Russia;
[2] National Research Center, "Kurchatov Institute", Akademika Kurchatova pl. 1, 123182 Moscow, Russia;
* Correspondence: kvassil@vega.protres.ru; Tel.: +7 4967 318232

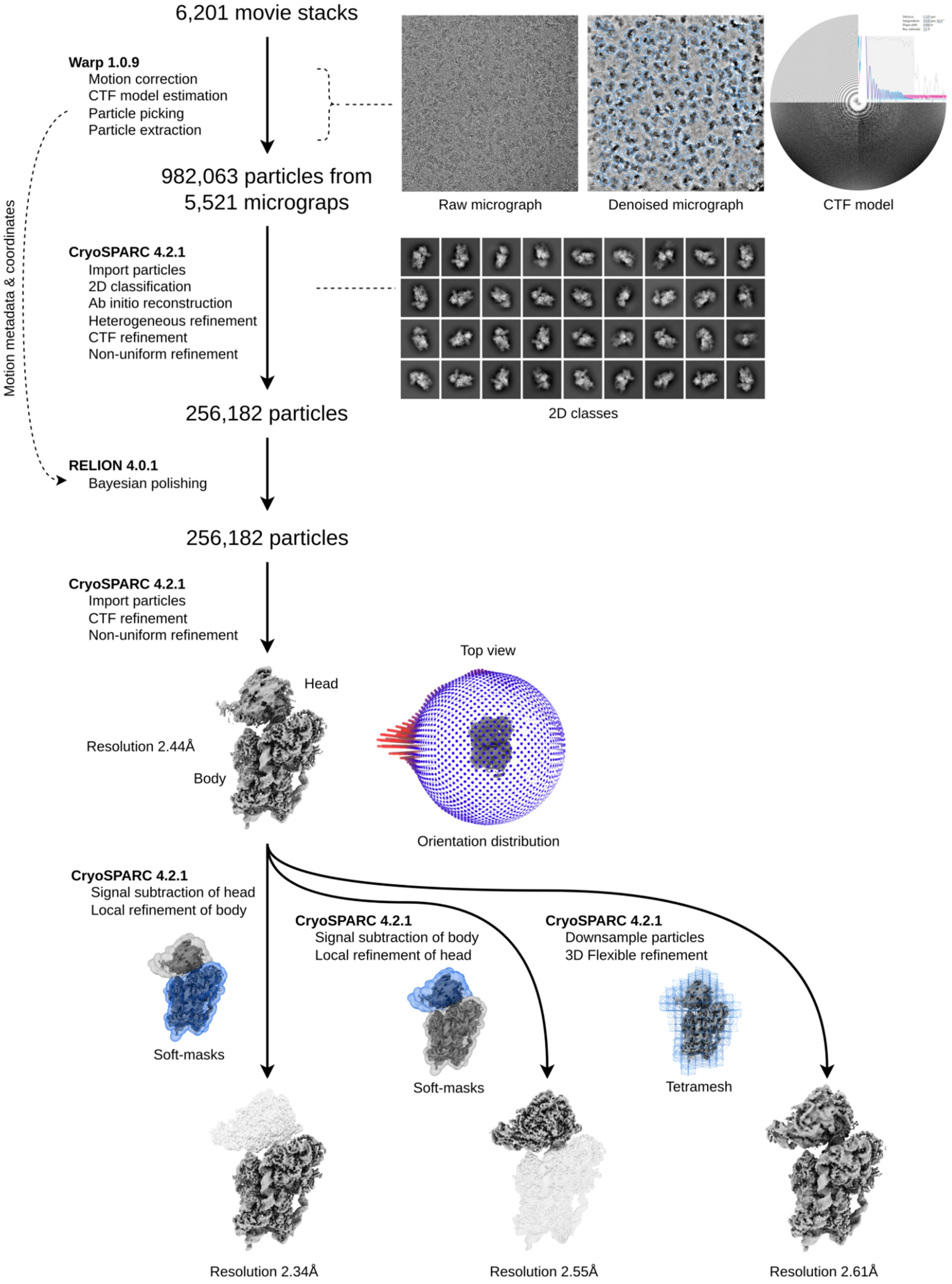

Supplementary Figure S1: Cryo-EM data processing scheme. At the top – a typical raw micrograph with the estimated -1.2 um defocus, its denoised variant with picked particles and 50x scaled motion tacks, the corresponding CTF model and final 2D class averages are shown. In the middle – the resulting 2.44 Å consensual map and the relevant orientation distribution plot are presented (generated using scripts from the UCSF pyem collection [6]). At the bottom – masking and local refinement of the head and body domains; 3D Flexible refinement of the whole 40S subunit map.

Supplementary Table S1: Cryo-EM data collection, processing, and model refinement statistics.

	40S body domain	40S head domain	3DFlex reconstruction
**Data collection**			
Grids		QF R1.2/1.3 300 mesh + 2 nm Ultrathin Carbon	
Microscope		TFS Titan Krios	
Detector		TFS Falcon II	
Voltage, *kV*		300	
Nominal magnification		75,000x	
Nominal defocus range, *μm*		-1.8 to -0.6	
Calibrated pixel size, *Å*		0.86	
Total exposure time, *s*		1.6	
Number of frames per stack		32	
Electron dose per frame, $e^{-}/Å^{2}$		2.6	
Number of micrographs (initial)		6,201	
**Data processing**			
Number of micrographs (used)		5,521	
Box size, *px*	480	480	440
Number of particles (collected)	982,063	982,063	982,063
Final number of particles	256,182	256,182	256,000
Resolution			
Final map $FSC_{0.143}$ resolution, *Å*	2.34	2.55	2.68
Map sharpening B-factor, $Å^{2}$	-76.0	-86.6	-73.6
Local resolution 1st and 3rd quartile, *Å*	2.55, 5.32	2.74, 5.33	2.75, 5.70
**Refinement**			
Initial model used	7qix	7qiy	
Model composition			
Non-hydrogen atoms	49016	25048	
Protein residues	2849	1878	
Nucleotides	1177	458	
Waters	1067	357	
Metal ions	47	28	
B-factors, $Å^{2}$			
Protein	73.58	71.28	
RNA	123.71	90.07	
R.m.s. deviation			
Bond length, *Å*	0.004	0.005	
Bond angles, °	0.681	0.831	
**Validation**			
MolProbity score	1.72	2.23	
Clashscore	12	17	
Poor rotamers, %	0.95	1.00	
Ramachandran plot			
Favored, %	97.31	92.76	
Allowed, %	2.69	7.21	
Disallowed, %	0	0	

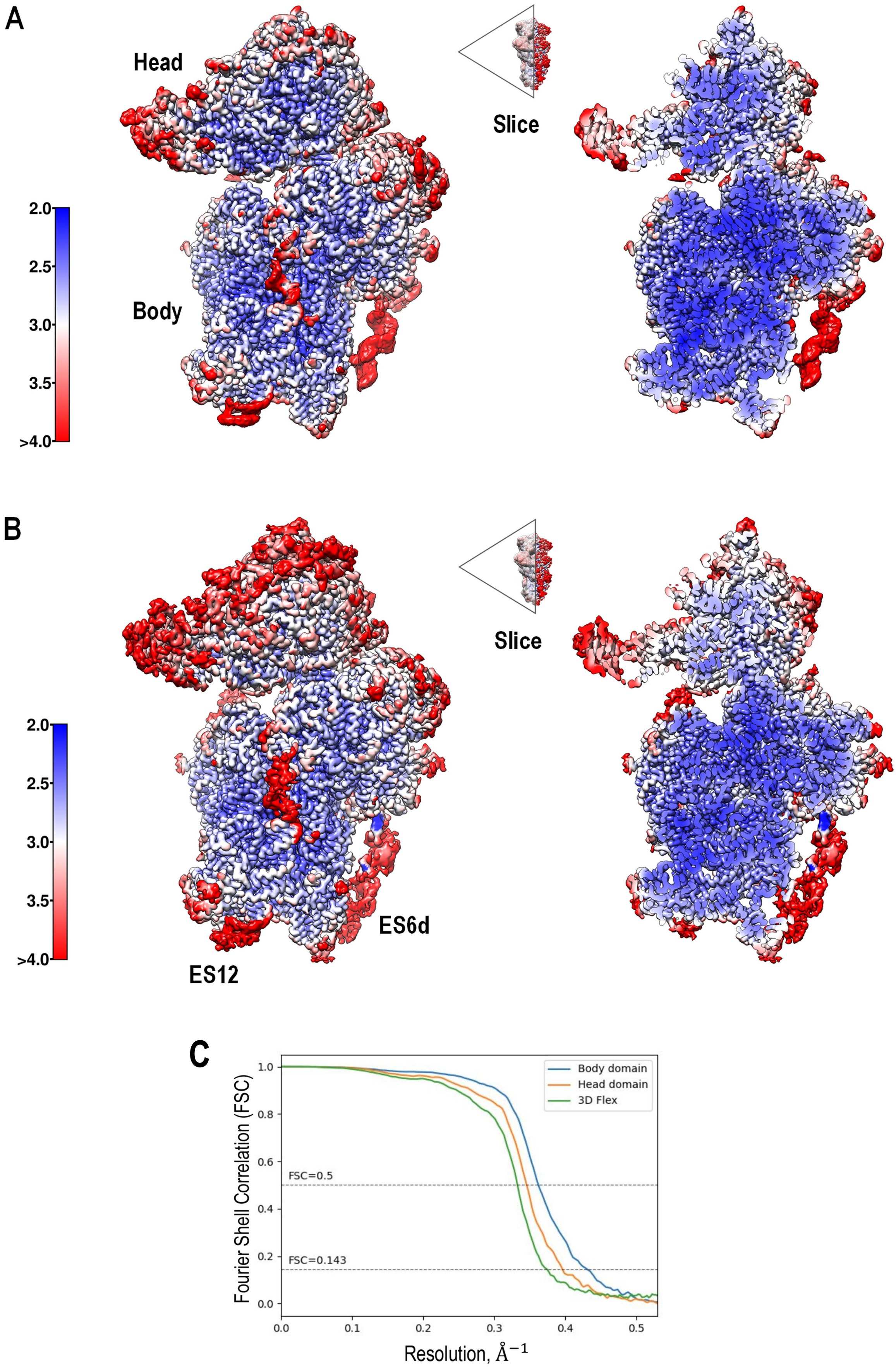

Supplementary Figure S2: Local resolution of wheat 40S subunit density maps. The intersubunit interface-side view of local resolution estimate for the plant 40S subunit maps obtained through local refinement of the head and body domains (A) and 3D Flexible refinement (B). Right panels – slice through the subunit, demonstrating high resolution of the stable internal regions. (C) Fourier shell correlation (FSC) plot between two half maps is shown for body, head and 3DFlex maps. Gold-standard FSC cut-off at 0.143 was used to estimate the final resolution.

>18

UACCUGGUUGAUCCUGCCAGUAGUCAU (A2M) UGCUUG (PSU) CU (OMC) AAAGAUUAAGCCAUGCAUGUGCAA
GUAUGAACCAAUUUGAACUGUGAAACUGCGAAUGGCUCAU (PSU) AAAUCAG (PSU) UAUAGUUUG (PSU) U (O
MU) GAUGGUACGUGCUACUCGGAUAACCGUAGUAAUUCUAG (A2M) GCUAA (OMU) ACGUGCAACAAACCCCGA
CUUCUGGGAGGGGCGCAUUUA (PSU) UAGAUAAAAGGCUGACGCGGGCUCUGCUCGCUGAUCC (OMG) AUGAUU
CA (PSU) GA (PSU) AACUCGACGGAUCGCACGGCCUUCGUGCCGGCGACGCAUCA (PSU) UCAAA (PSU) U (PS
U) CUGCCCUAUCAACUUUCGAUGGUAGGAUAGGGGCCUACCAUGGUGGUGACGGG (PSU) GACGGAGAAUUAGG
GUUCGAUUCCGGAGA (OMG) GGAGCCUGAGAAACGGCUACCACAU (OMC) CAAGG (A2M) AGGCAGCAG (OMG)
CGCGC (A2M) AAUUACCCAAUCCUGACACGGGGAGGU (A2M) GUGACAAUAAAUAACAAUACCGGGCGCAUUAG
UGUCUGGUAAUUGGAAUGAGUACAAUCUAAAUCCCUUAACGAGG (A2M) UCCAUUGGAGGGCAAGUCUGGUGCC
AGCAGCCGCGGUAAUUCCAGCUCCAAUA (OMG) CGUA (UY1) A (PSU) (PSU) UAAGUUG (OMU) UGCAGUU (A
2M) AAAAGCUCGUAG (PSU) UGGACCUUGGGCCGGGUCGGCCGGUCCGCCUCACGGCGAGCACCGACCUACUCG
ACCCUUCGGCCGGCAUCGCGCUCCUAGCCUUAAUUGGCCGGGUCGUGUUUCCGGCAUCGUUACUUUGAAGAAA (
PSU) (PSU) AGAGUGCUCAAAGCAAGCCAUCGCUCUGGAUACAUU (A2M) GCA (PSU) GGGA (PSU) AACAUCA
UAGGAUUCCGGUCCUAUUGUGUUGGCCUUCGGGAUCGGAGUAAUGAUUAAUAGGGACAGUCGGGGGCAUUCGUA
UUUCAUAGUCAGAGGUGAAAUUCUUGGAUUUAUGAAAGACGAACAACUGCGAAAGCAUUUGCCAAGGAUGUUUU
CAUUAAUCAAGA (A2M) CGAAAGUUGGGGGCUCGAAGACGA (PSU) CAGAUACCG (OMU) CCUAGUCUCAACCA
UAAACGAUGCCGACCAGGGAUCGGCGGAUGUUGCUUAUAGGACUCCGCCGGCACC (PSU) UAUGAGAAAUCAAA
G (PSU) CUUUGGG (PSU) UCCGGGGGGAGUA (PSU) GGUCGCAA (OMG) GCUGAAACUUAAAGGAAUUGACGGA
AGGCACCACCAGGCGUGGAGCC (PSU) GCGGCUUAAUU (PSU) GACUCAACACGGGGAAACUUACCAGG (PSU)
(OMC) CAGACAUAGCAAGGA (OMU) UGACAGACUGAGAGCUCUUUCUUGAUUC (OMU) A (OMU) GGGUGG (OMU
) G (OMG) UGCAUGGC (4AC) GUUCUUAGU (PSU) GGUG (OMG) AGCGA (PSU) UUG (PSU) CUGG (PSU) UAAU
UCCGUUAACGA (A2M) CGAGA (OMC) CUCAGCCUGCUAACUAGCUAUGCGGAGCCAUCC (OMC) (PSU) CCGCA
GCUAGCU (OMU) CUUAGAGGGACUAUCGCCGUU (PSU) AGGCGACGGAAGUUUGAGGCAAUAACA (OMG) GUCU
G (PSU) GAUGCCC (OMU) UAGAUGUUCUGGGCCGCACGCGCGCUACAC (PSU) GAUGUAUUCAACGAGUAUAUA
GCCUUGGCCGACAGGCCCGGGUAAUCUUGGGAAA (PSU) U (PSU) CAUCGUGAUGGGGAUAGAUCAUUGCAAUU
GU (PSU) GGUCUUCAACG (A2M) G (7MG) AAUGCCUAGUAAGCGCGAGUCA (PSU) CAGC (PSU) CGCGU (PSU
) GACUACGUCCCUGCCCUU (PSU) GUACACACCG (OMC) CCGUCGCUCCUACCGAUUGAAUGGUCCGGUGAAGU
GUUCGGAUCGCGGCGACGGGGGCGGUUCGCCGCCCCCGACGUCGCGAGAAGUCCAUUGAACCUUAUCAUUUAGA
GGA (A2M) GGAGAAGUCGUA (6MZ) CAAGGUUUC (4AC) GUAGGUG (MA6) (MA6) CCUGCGGAAGGAUCAUUG

Supplementary Figure S3: ***T. aestivum*** **18S rRNA sequence best fitted into the cryo-EM density map.** GeneBank ID XR_006452643.1. Tree-letter PDB codes for the modifications are used.

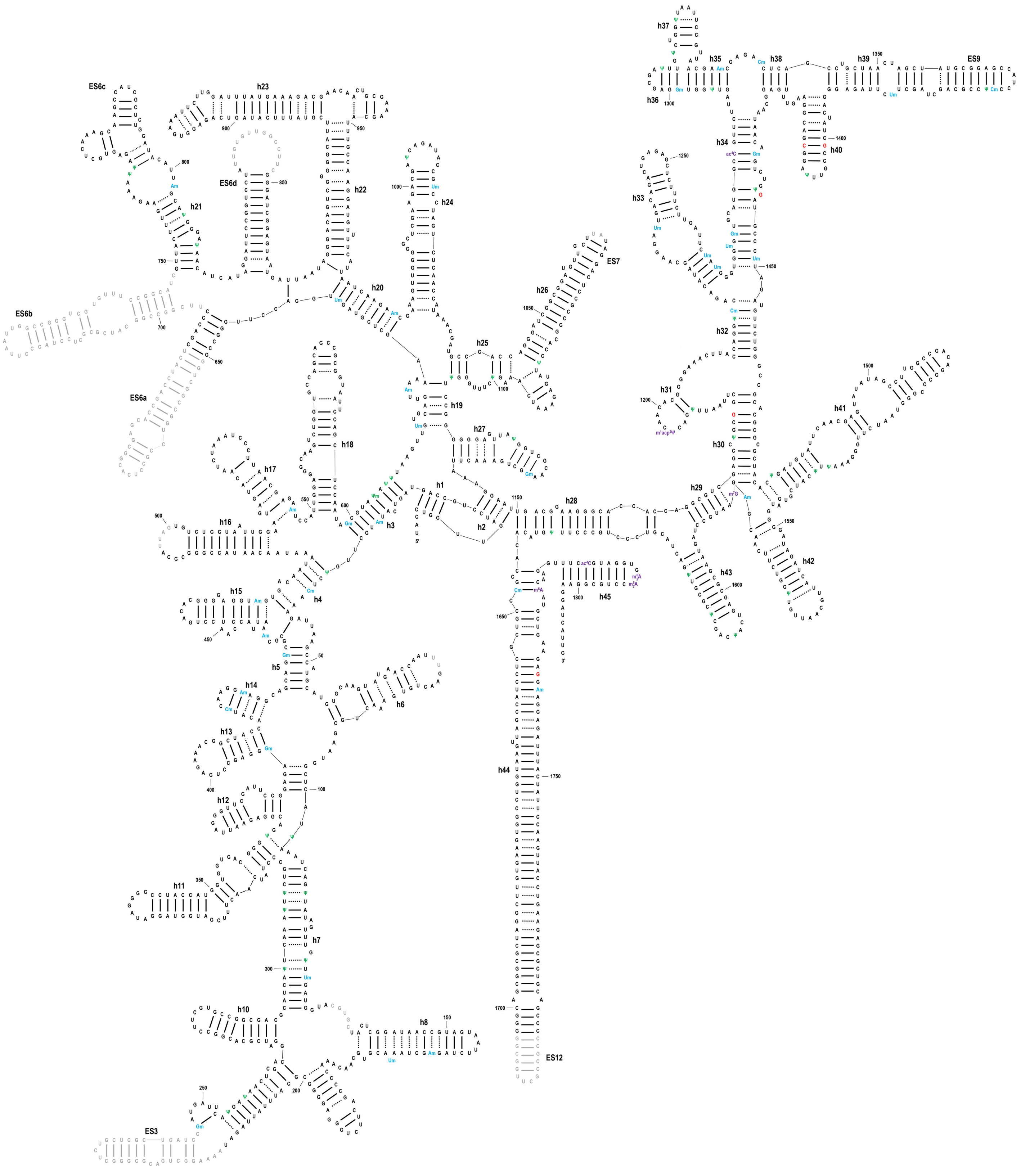

Supplementary Figure S4: Secondary structure diagram for the *Triticum aestivum* 18S rRNA based on the cryo-EM analysis. Regions poorly resolved in the density maps appear in grey. Non-canonical base-pair interactions are marked as dashes. 2′-O-methylations are highlighted in blue, pseudouridines – in green, and conservative auxiliary base modifications – in magenta.

Supplementary Table S3: 2'-O-Methylation of wheat 18S rRNA

T.aestivum [a]	**Verified** [b]	***S. lycopersicum*** [c]	**snoRNA** [d] ***A.thaliana***	**Human** [e]	**Yeast** [e]
**Am28**	[1, 2]	Am28	U27-2	Am27	Am28
**Cm38**	[1, 2]	Cm38	snoR66		
**Um123**	[1, 2]	Um123	snoR116	Um121	
**Am162**	[1, 2]	Am162	snoR18	Am166	
**Um168**		U168	snoR122	Um172	
U213	[2]	U213	SnoR65		
**Gm246**	[1, 2]	Gm246	snoR124		
**Gm392**	[1, 2, 3]	Gm392	snoR30	Gm436	
G393	[3]	G393	snoR58		
**Cm418**	[1, 2]	Cm418	U14a/b	Cm462	Cm414
**Am424**		A424			
**Gm434**		G434			
**Am440**	[1, 2, 3, 4]	Am440	snoR15	Am484	Am436
**Am468**	[1, 2, 4]	Am468	snoR17	Am512	
C473	[1, 2, 4]	Cm473	snoR7	Cm517	
**Am545**	[1, 2, 3]	Am544	snoR41Y	Am590	Am541
U582	[1, 2]	Um581	snoR77Y	Um627	Um578
**Gm599**	[1, 2]	Gm598	U54	Gm644	
**Ψm604**	[1, 2]	Ψm603	snoR115		
G611	[3]	G610	snoR62		
**Um615**	[1, 2]	Um614	snoR13		
**Am623**	[1, 2]	Am622	U36	Am668	Am619
A781	[2]	A779			
A797	[2]	A795			
**Am802**	[1, 2, 4]	Am800			Am796
A812	[2]	A810			
**Am979**	[1, 2, 4]	Am977	snoR59	Am1031	Am974
**Um1014**	[1, 2, 3, 5]	Um1012	snoR20-1		
C1015	[5]	C1013	snoR20-2		
**Gm1131**		G1129			
A1189	[2]	A1187			
**Cm1220**	[1, 2]	Cm1218	SnoR166	Cm1272	
**Um1236**	[1, 2]	U1234	snoR14	Um1288	
**Um1265**	[1]	Um1263	snoR67		
**Um1267**	[1, 2, 5]	Um1265	snoR32		
**Um1274**	[1, 2, 4, 5]	Um1272	snoR34	Um1326	Um1269
G1275	[3]	G1273	snoR26		
**Gm1276**	[1, 2, 4, 5]	Gm1274	snoR21a	Gm1328	Gm1271
**Gm1300**		G1298			
**Am1331**	[1, 2]	Am1328	snoR32	Am1383	
**Cm1337**		C1334			
**Cm1371**		C1368			
**Um1385**	[1, 2]	Um1382	U61	Um1442	
**Gm1435**	[1, 2]	Gm1432	snoR19	Gm1490	Gm1428
**Um1449**	[1, 2, 3]	Um1446	snoR19		
U1557	[2]	U1553			
**Am1582**	[1, 2]	Am1578	snoR8		
C1633	[3]	C1629	snoR70		
**Cm1648**	[1, 2]	Cm1645	U43	Cm1703	Cm1639
**Am1761**	[1, 2, 3]	Am1758	snoR23		

[a] 2'-O-Methylation of wheat 18S rRNA determined through the analysis of the cryo-EM density map; [b] Published biochemical data on the 2'-O-Methylation of a given nucleotide in plants; [c] 2'-O-Methyl groups in the model of tomato 18S rRNA checked by mass-spectrometry analysis [1]; [d] Published *Arabidopsis thaliana* plant snoRNAs that code for the corresponding modification; [e] 2'-O-Methyl groups in human and yeast 18S rRNAs located at the relevant positions; Green coloring indicates *T.aestivum* rRNA modifications not detected in *S lycopersicum* structure. Red coloring – unmodified nucleotides reported to be methylated in *S lycopersicum*.

Supplementary Table S4: Pseudouridinilation of wheat 18S rRNA

T.aestivum[a]	Verified[b]	S. lycopersicum[c]	snoRNA[d] A.thaliana	Human[e]	Yeast[e]
**Ψ35**	[6]	U		Ψ34	
**Ψ103**	[1]	Ψ103			
**Ψ111**	[1, 6]	Ψ111	snoR100	Ψ109	
**Ψ121**	[1, 6]	Ψ121		Ψ119	Ψ120
**Ψ208**	[1]	Ψ208			
**Ψ255**	[1, 6]	Ψ255		Ψ296	
**Ψ258**	[1, 6]	Ψ258			
**Ψ300**	[1]	Ψ300			
**Ψ308**		U308			
**Ψ306**	[1, 6]	Ψ306			Ψ302
**Ψ362**	[1]	Ψ362	snoR86	Ψ406	
U383	[1, 6]	Ψ383			
U451	[1]	Ψ451			
U585	[1, 6]	Ψ584			
**Ψm604**	[1]	Ψm603			
**Ψ606**	[1, 6]	Ψ605	snoR91	Ψ654	
**Ψ607**		U606			
**Ψ636**	[1, 6]	Ψ635	snoR73	Ψ681	Ψ632
U755	[1]	Ψ753	snoR77	Ψ801	
**Ψ764**	[1, 6]	Ψ762	snoR91	Ψ814	Ψ759
**Ψ765**	[6]	U763	snoR139	Ψ815	
**Ψ806**	[6]	U804	snoR99		
**Ψ811**	[1]	Ψ809			
U914	[1]	Ψ912			
U951	[1, 6]	Ψ949			
**Ψ952**	[1, 6]	Ψ950	snoR90	Ψ1004	

T.aestivum	Verified	S. lycopersicum	snoRNA A.thaliana	Human	Yeast
**Ψ1004**	[1, 6]	Ψ1002	snoR5	Ψ1056	Ψ999
U1029	[1, 6]	Ψ1027		Ψ1081	
**Ψ1084**		U1082			
**Ψ1100**		U1098			
**Ψ1108**	[1, 6]	Ψ1106	snoR134		
**Ψ1122**	[1, 6]	Ψ1120	snoR5	Ψ1174	
**Ψ1180**	[1]	Ψ1178			
**Ψ1186**	[1]	Ψ1184			
**Ψ1192**	[1]	Ψ1190	snoR161	Ψ1244	Ψ1187
U1212	[1, 6]	Ψ1210	snoR137		
**Ψ1219**	[1, 6]	Ψ1217	snoR140		
**Ψ1295**	[1]	Ψ1292			
**Ψ1306**	[1, 6]	Ψ1303	snoR88		
**Ψ1310**		Ψ1307	snoR160		
**Ψ1315**	[1, 6]	Ψ1315		Ψ1367	
**Ψ1372**		U1369			
**Ψ1407**		U1404			
**Ψ1441**		U1438			
**Ψ1480**		U1477			
U1487	[1, 6]	Ψ1484	U1483		
**Ψ1536**		U1532			
**Ψ1538**	[1, 6]	Ψ1534	snoR152		
U1541	[1]	Ψ1537			
**Ψ1570**	[1]	Ψ1566			
**Ψ1607**		U1637			
**Ψ1637**	[1, 6]	Ψ1633		Ψ1692	

[a] Pseudouridines in wheat 18S rRNA determined through the analysis of the cryo-EM density map; [b] Published biochemical data on the pseudouridinilation of a given nucleotide in plants; [c] Pseudouridines in the model of tomato 18S rRNA checked by mass-spectrometry analysis [1]; [d] Alleged *Arabidopsis thaliana* plant snoRNAs that code for the corresponding modification; [e] Pseudouridines in human and yest 18S rRNAs located at the relevant positions; Green coloring indicates *T.aestivum* rRNA modifications not detected in *S lycopersicum* structure. Red coloring – unmodified nucleotides that reported to be pseudouridinilated in *S lycopersicum*.

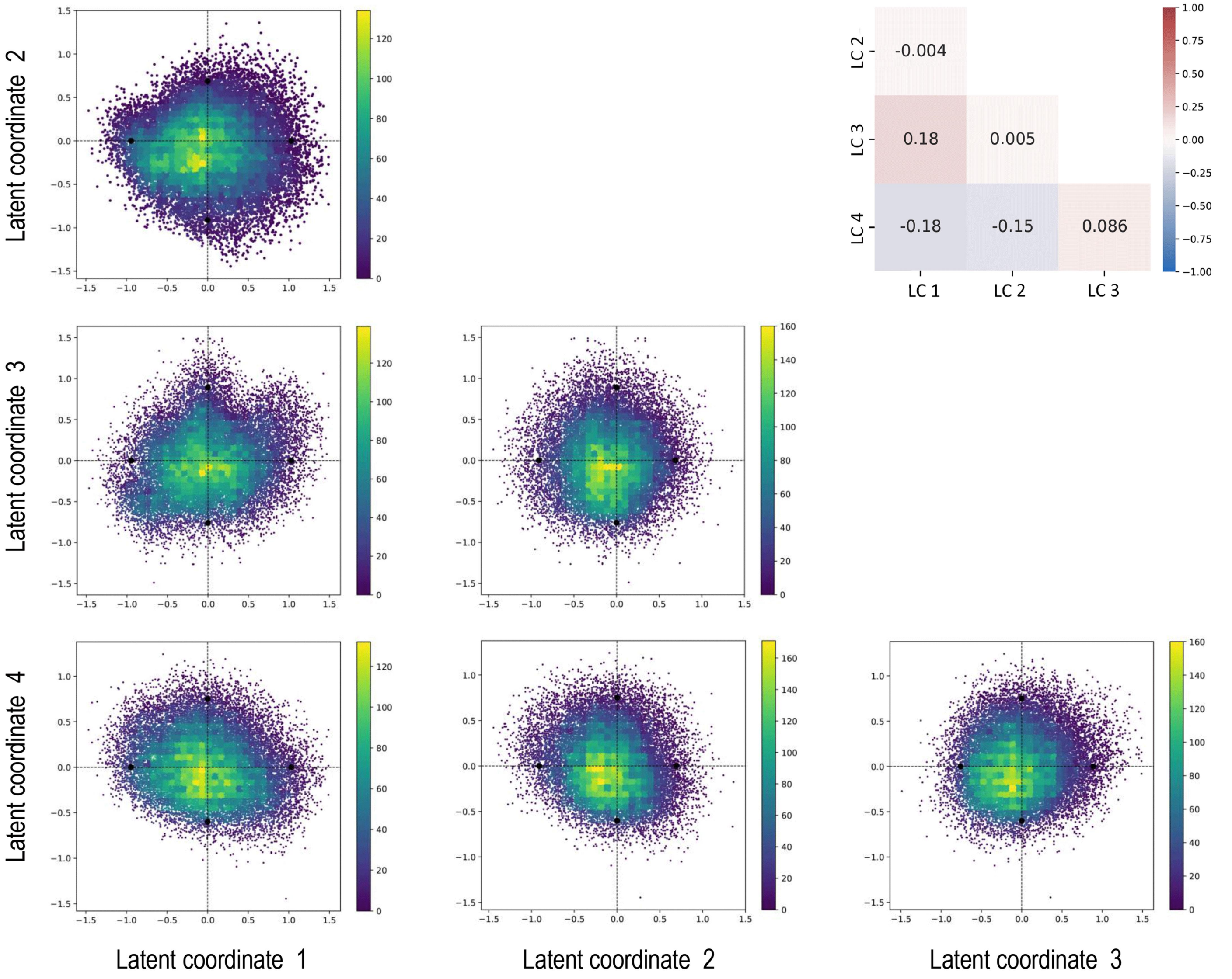

Supplementary Figure S5: Correlation between major motions inside flexible structure of 40S ribosomal subunit. Scatter plots showing the distribution of particle latent coordinates across the dataset. Data point color indicates a number of particles adopting a specific conformation. Top-right panel – the matrix of pair correlations between the latent coordinate sets.

References

1. Cottilli, P.; Itoh, Y.; Nobe, Y.; Petrov, A. S.; Lisón, P.; Taoka, M.; Amunts, A. Cryo-EM structure and rRNA modification sites of a plant ribosome. *Plant communications* **2022**, *3*, 100342.

2. Azevedo-Favory, J.; Gaspin, C.; Ayadi, L.; Montacié, C.; Marchand, V.; Jobet, E.; ... Sáez-Vásquez, J. Mapping rRNA 2'-O-methylations and identification of C/D snoRNAs in Arabidopsis thaliana plants. *RNA biology* **2021**, *18*, 1760-1777.

3. Barneche, F.; Steinmetz, F.; Echeverrıa, M. Fibrillarin Genes Encode Both a Conserved Nucleolar Protein and a Novel Small Nucleolar RNA Involved in Ribosomal RNA Methylation inArabidopsis thaliana. *Journal of Biological Chemistry* **2000**, *275*, 27212-27220.

4. Brown, J.W.; Clark, G.P.; Leader, D. J.; Simpson, C.G.; Lowe, T.O.D D. Multiple snoRNA gene clusters from Arabidopsis. *RNA* **2001**, *7*, 1817-1832.

5. Liang-Hu, Q.; Qing, M.; Hui, Z.; & Yue-Qin, C. Identification of 10 novel snoRNA gene clusters from Arabidopsis thaliana. *Nucl. Acids Res.* **2001**, *29*, 1623-1630.

6. Sun, L.; Xu, Y.; Bai, S.; Bai, X.; Zhu, H.; Dong, H.; ... & Song, C. P. Transcriptome-wide analysis of pseudouridylation of mRNA and non-coding RNAs in Arabidopsis. *Journal of Experimental Botany* **2019**, *70*, 5089-5600.
